# Effect of Powder Heat Treatment on Chemical Composition and Thermoelectric Properties of Bismuth Antimony Telluride Alloys Fabricated by Combining Water Atomization and Spark Plasma Sintering

**DOI:** 10.3390/ma14112993

**Published:** 2021-06-01

**Authors:** Dong-won Shin, Peyala Dharmaiah, Jun-Woo Song, Soon-Jik Hong

**Affiliations:** Division of Advanced Materials Engineering, Institute for Rare Metals, Kongju National University, 275, Budae-dong, Cheonan City 330-717, Chungcheongnam-do, Korea; clever_05@naver.com (D.-w.S.); dharmaphysics@gmail.com (P.D.); sipmirri@naver.com (J.-W.S.)

**Keywords:** water atomization, bismuth antimony telluride, spark plasma sintering, heat treatment, thermoelectric materials

## Abstract

In this work, Bi_0_._5_Sb_1_._5_Te_3_ materials were produced by an economically viable and time efficient water atomization process. The powder samples were heat treated at different temperatures (673 K, 723 K, 743 K, 773 K, 803 K, and 823 K) followed by spark plasma sintering (SPS). It was found that the Te evaporated slightly at 723 K and 743 K and became dominated at 773 K, 803 K, and 823 K, which severely influences the thermoelectric properties. The electrical conductivity was significantly improved for over 803 K heat treated samples due to the remarkable improvement in hole concentration. The power factor values for the 803 K and 823 K samples were significantly larger at T > 350 K compared to other samples. Consequently, the peak ZT of 0.92 at 350 K was obtained for the 803 K sample, which could be useful in commercial thermoelectric power generation.

## 1. Introduction

Thermoelectric (TE) materials enable the direct conversion of waste heat to electricity and vice versa for power generation and cooling [1,2]. Regardless of conversion efficiency, TE materials are gaining a lot of attention for green energy conversion. The efficiency of TE materials is determined by the dimensionless parameter called figure of merit, *ZT = S^2^σT/κ*, where *S* is the Seebeck coefficient, *σ* is the electrical conductivity, *κ* is the total thermal conductivity comprised of carrier contribution (*κ_el_*) and lattice contribution (*κ_L_*), and *T* is the absolute temperature. Therefore, enhancement of *ZT* is not an easy task due to strong interdependency between these parameters (*S*, *σ,* and *κ*) through carrier concentration. For the past decades, significant progress has been made in improvement of *ZT* by reduction of thermal conductivity through nano-compositing [3,4], twin boundary engineering [5], hierarchical structuring [6] and increasing the power factor (*S^2^σ*) via defect engineering [7] and band structure engineering [8].

Bi–Sb–Te based alloys are reviewed to demonstrate the high potential commercialized p-type thermoelectric materials at room temperature due to their outstanding TE properties. In recent years, there have been lots of endeavors towards property optimization and microstructural modification to enhance the figure of merit in Bi–Sb–Te alloys through the powder metallurgy process. For instance, nanostructure powder materials are produced through high energy ball milling [9,10], melt-spinning [11], or solvothermal/hydrothermal process [12,13] and then subsequently compacted by hot pressing (HP) or spark plasma sintering. However, these fabrication methods were effective for enhancement of *ZT* by reducing *κ_L_* through phonon scattering, but have low productivity and are difficult to apply in commercial applications. Recently, we developed a simple and economical viable water atomization method for the fabrication of alloy powders [14], though water atomized powder was required as further treatment for removing oxygen on the surface of the powder, because the molten stream directly interacts with water. The oxygen impurities severely affect the electrical conductivity in Bi_0_._5_Sb_1_._5_Te_3_ alloys [15,16]. Pre-heat treatment or annealing can be applied to remove the oxygen from thermoelectric powders.

In this study, the effects of various heat treatment temperatures on water atomized Bi–Sb–Te thermoelectric powder, followed by sintering, were examined. We discuss the thermoelectric transport mechanisms correlated with chemical composition and microstructure of heat treated Bi–Sb–Te samples to enhance the figure of merit (*ZT*).

## 2. Experimental Procedure

### 2.1. Sample Preparation

As in our previous work, we used high purity elements of Bi (99.99%), Sb (99.9%), and Te (99.99%) for the fabrication of Bi_0_._5_Sb_1_._5_Te_3_ thermoelectric alloy powder using the water atomization (WA) process [14]. The water atomized thermoelectric powder was loaded into a quartz tube and heat-treated at 673 K, 723 K, 743 K, 773 K, 803 K and 823 K for 60 min in a vacuum furnace of 10^−2^ torr. The heat-treated powders were consolidated by a spark plasma sintering (SPS) method applied at 673 K for 10 min under a uniaxial pressure of 50 MPa in a vacuum of 10^−2^ torr.

### 2.2. Characteristics of the Heat-Treated Thermoelectric Powder and Bulks

The phase constituents of the thermoelectric powders and bulks were identified by X-ray diffraction (XRD, CuKα, MiniFlex-600, Rigaku, Tokyo, Japan) in the 2θ range of 20–70 degrees at room temperature. The quantitative chemical composition of powder and bulk samples were analyzed by X-ray fluorescence spectrometer (XRF, SEA2220A, SII Nano Technology Inc., Chiba, Japan). The cross-sectional microstructure of the powder and bulks was observed by field emission scanning electron microscopy (FESEM, MIRA LMH, TESCAN, Brno, Czech Republic). The bulk samples were carefully cut into rectangular pieces with a dimension of 4 × 4 × 12 mm^3^ using a wire cutting machine. Then the temperature dependent Seebeck coefficient, electrical conductivity and power factor were measured by the four-probe method (TEP-1000, Seepel, Hwaseong, South Korea) in the temperature range of 300–500 K. The thermal conductivity was calculated by the equation of κ = λC_p_d, where λ is the thermal diffusivity measured on the disk-shaped (<12.7 mm in diameter with <2 mm thick) specimen by laser flash method (LFA 457, Netzsch, Selb, Germany), C_p_ is the specific heat capacity measured by differential scanning calorimeter (DSC-8000, PerkinElmer, Waltham, MA, USA), and d is the actual density of the bulk sample measured by Archimedes method. The carrier concentration and mobility were measured by a hall measurement setup (HMS-3000, Ecopia, Anyang, South Korea) under a magnetic field of 0.55 T.

## 3. Results and Discussion

**Figure 1:**
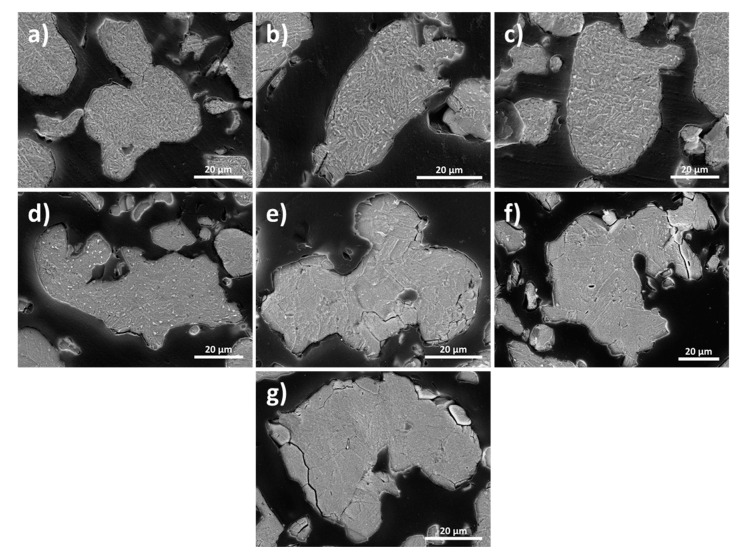
SEM cross-sectional microstructure of the (**a**) water atomized (WA) powder, heat treated at (**b**) 673 K, (**c**) 723 K, (**d**) 743 K, (**e**) 773 K, (**f**) 803 K, and (**g**) 823 K.

Figure 1 shows the cross-sectional microstructure of the (a) water atomized (WA) powder, heat treated at (b) 673 K, (c) 723 K, (d) 743 K, (e) 773 K, (f) 803 K, and (g) 823 K. It was observed that the microstructure of all powder samples showed needle-like grains and their length and width increased with increasing heat treatment temperature. The average grain size of WA powder was about 1–3 µm and this was increased to 10–13 µm for 823 K, which would severely influence the final properties of the materials.

**Figure 2:**
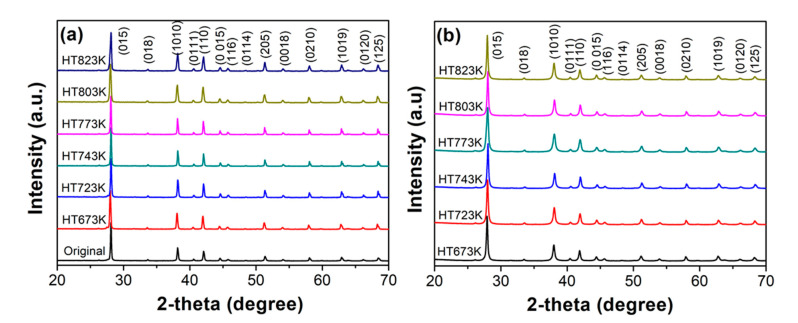
X-ray diffraction patterns of Bi_0_._5_Sb_1_._5_Te_3_ (**a**) powder, and (**b**) bulk samples obtained using heat treated powder at different temperatures.

Figure 2 shows the X-ray diffraction patterns of the (a) water atomized (WA) powder, heat treated at 673 K, 723 K, 743 K, 773 K, 803 K, and 823 K and the bulk samples (b) obtained after SPS. The diffraction patterns of powder and bulk samples can be well coincided with the rhombohedral Bi_0_._5_Sb_1_._5_Te_3_ (JCPDS#491713) phase. There are no other impurities detected within the detection limit of the XRD, indicating the successful fabrication of single phase Bi_0_._5_Sb_1_._5_Te_3_ alloys. The relative densities of all the sintered samples exhibited more than 99% of its theoretical density.

**Figure 3:**
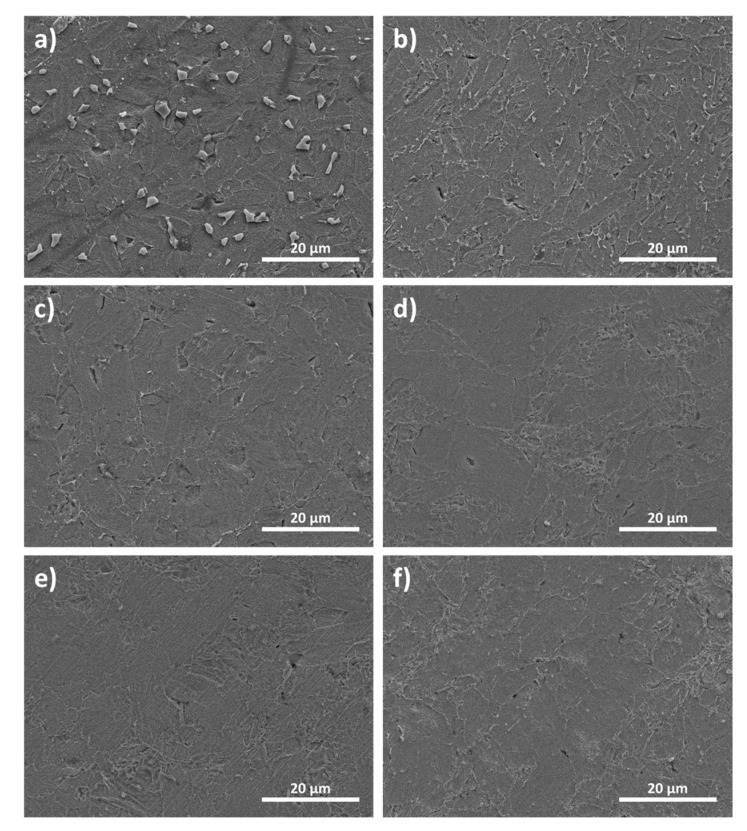
SEM cross-sectional micrographs of spark plasma sintered bulk samples obtained using heat treated powder at different temperatures: (**a**) 673 K, (**b**) 723 K, (**c**) 743 K, (**d**) 773 K, (**e**) 803 K, and (**f**) 823 K.

Figure 3 shows the cross-sectional microstructure of bulk samples obtained using WA powder heat treated at 673 K, 723 K, 743 K, 773 K, 803 K, 823 K and following consolidation by SPS. It can be seen from the micrographs that the powder was fully dense with more random oriented grains and grain boundaries. The average grain size increased by about 4 µm, 7 µm, 9 µm, 8 µm, 10 µm and 12 µm for 673 K, 723 K, 743 K, 773 K, 803 K, and 823 K samples, respectively. In addition, the white particles were observed and identified as a tellurium (Te) at 673 K, which decreased significantly with increasing heat treatment temperature. Based on the results, heat-treatment can severely affect the chemical composition of the Bi_0_._5_Sb_1_._5_Te_3_ alloys.

To study the influence of powder heat treatment on chemical composition, the X-ray fluorescence spectrometer (XRF) was utilized, and the results are shown in Table 1. The Te evaporated slightly at 723 K and 743 K and becomes dominated at 773 K, 803 K and 823 K. Thus, the Te content decreased with increasing heat treatment temperature.

**Figure 4:**
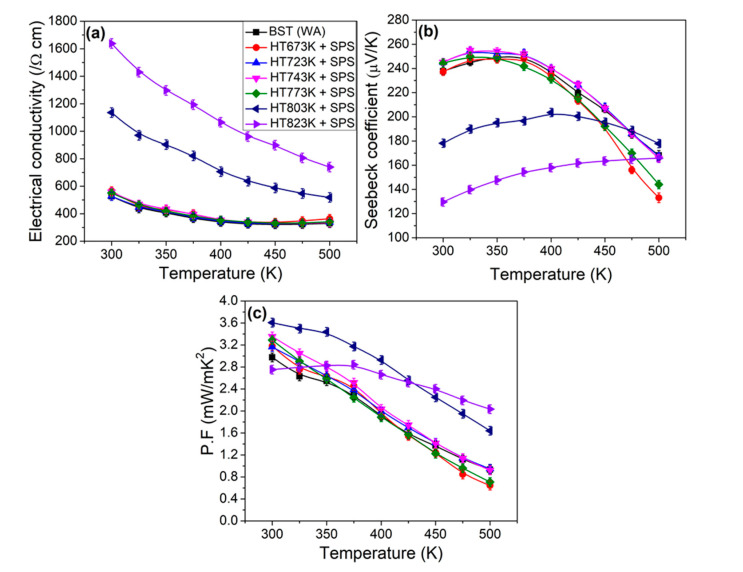
Temperature dependence of electrical transport properties for the bulk samples obtained by powder heat treatment at different temperatures and consolidation: (**a**) Electrical conductivity (*σ*), (**b**) Seebeck coefficient (*S*), and (**c**) Power factor (*PF*).

Temperature dependent (a) electrical conductivity, (b) Seebeck coefficient, and (c) power factor for all bulk samples are shown in Figure 4. The trends in electrical conductivity for all the bulk samples are similar, so that *σ* decreased with increasing temperature indicating a metallic transport behavior. It is found that the electrical conductivity for the 673 K, 723 K, 743 K, 773 K samples exhibited similar values, while the 803 K and 823 K samples showed larger electrical conductivity. It is known that the electrical conductivity is directly proportional to carrier concentration (*n*), and mobility (*µ*) can be represented by *σ = neµ*, where *e* is the charge of an electron. As shown in Table 2, the hole concentration increased with increase of heat treatment temperature. In particular, the powder samples heat treated at 803 K and 823 K samples have significantly higher carrier concentration than other samples. The carrier concentration is sensitive to the chemical composition in Bi_0_._5_Sb_1_._5_Te_3_ alloys. The literature shows that evaporation of *Te* causes increased formation of anti-structure defects via the occupation of *Te* sites with *Bi* or *Sb* atoms, which leads to increased hole concentration, according to the following equations [17,18,19].

(1) $$Bi_{2}Te_{3}=2Bi_{Te}^{\prime }+2V_{Bi}^{\prime }+V_{Te}+\left(\frac{3}{2}\right)Te_{2}\left(g\right)+2h,\;$$

(2) $$Sb_{2}Te_{3}=2Sb_{Te}^{\prime }+2V_{sb}^{\prime }+V_{Te}+\left(\frac{3}{2}\right)Te_{2}\left(g\right)+2h,\;$$

where $V_{Te}$, $V_{Bi}^{\prime }$ and $V_{Sb}^{\prime }$ are the *Te*, *Bi*, and *Sb* vacancies, respectively. This significant rise in hole concentration may be the reason for the enhancement of electrical conductivity.

Figure 4b shows the temperature dependent Seebeck coefficient (*S*) for all the bulk samples. The Seebeck coefficient for the 673 K, 723 K, 743 K, 773 K samples exhibited a similar tendency with increased temperature and reached a maximum value at 350 K, while the maximum Seebeck coefficient shifted to high temperatures for the 803 K and 823 K samples. In addition, the Seebeck coefficient for the 673 K, 723 K, 743 K, 773 K samples exhibited similar values, while the 803 K and 823 K samples showed a smaller Seebeck coefficient. This behavior is similarly observed in the variation of electrical conductivity in the bulk samples. Assuming a single parabolic band model (SPB), the relation between the density of states effective mass (m*), Seebeck coefficient and hole carrier concentration (*n*) can be described as [5].

(3) $$S=\frac{k_{B}}{e}\left[\frac{\left(\frac{5}{2}+\lambda \right)F_{\frac{3}{2}+\lambda }\left(\eta \right)}{\left(\frac{3}{2}+\lambda \right)F_{\frac{1}{2}+\lambda }\left(\eta \right)}-\eta \right]\;$$

(4) m*=h22kBT(n4πF12(η))23     

(5) $$F_{i}\left(\eta \right)=\int_{0}^{\infty }\frac{x^{i}}{1+e^{x-\eta }}dx\;$$

(6) $$\eta =\frac{E_{f}}{k_{B}T}$$

where *E_f_* is the Fermi energy, *η* is the reduced Fermi level, *F_i_*(*η*) is the Fermi integral, *k_B_* is the Boltzmann constant, *h* is the Planck’s constant, *λ* is the scattering factor considered to be −0.5 for acoustic phonon scattering mode, and *e* is the electronic charge. The carrier effective mass m* was estimated according to Equations (3)–(6) and shown in Table 2. The carrier effective mass for all the samples was approximately unchanged. Therefore, the smaller Seebeck coefficient for the 803 K and 823 K samples was mainly due to the significant increase of *n.*

Figure 4c shows the temperature dependent power factor (*PF*) for all the bulk samples. The *PF* values increased with increase of heat treatment temperature. Interestingly, the *PF* values for the 803 K and 823 K exhibited significantly larger values at T > 350 K compared to other samples. A maximum power factor at 300 K was about 3.61 × 10^−3^ Wm^−1^K^−2^ for the 803 K samples, primarily owing to enhanced electrical conductivity.

**Figure 5:**
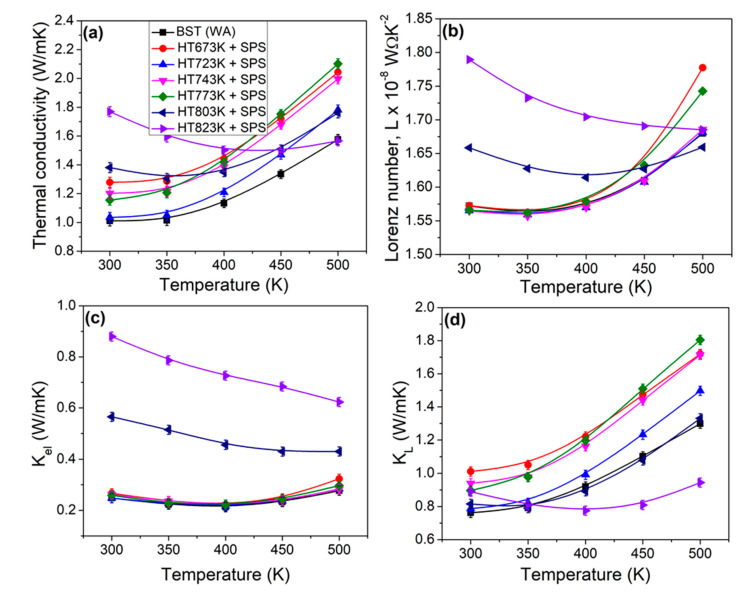
Temperature dependence of thermal transport properties for the bulk samples obtained by powder heat treatment at different temperatures and consolidation: (**a**) total thermal conductivity (*κ*), (**b**) Lorenz number (*L)*, (**c**) electronic thermal conductivity (*κ_el_*), and (**d**) lattice thermal conductivity (*κ_L_*).

Temperature dependent (a) total thermal conductivity, (b) electronic thermal conductivity and (c) lattice thermal conductivity for all the bulk samples are shown in Figure 5. The total thermal conductivity increased when increasing the heat treatment temperature as shown in Figure 5a. The trends in total thermal conductivity for the 673 K, 723 K, 743 K, 773 K samples gradually increase with increasing testing temperature, due to the increase of ambipolar thermal conductivity. However, the total thermal conductivity for the 803 K and 823 K samples first decreases due to the increasing phonon–phonon scattering, and then increases with the testing temperature. The onset of intrinsic excitation temperature is higher for the 803 K and 823 K samples than below 773 K of heat treatment. It is known that the total thermal conductivity is described according to *κ* *= κ_el_* *+ κ_L_*, where *κ_el_* is the electronic thermal conductivity obtained by the Wiedemann-Franz law, *κ_el_* *= LσT*; here *L* is the Lorenz number and can be estimated by the assumption of a single parabolic band model (SPB) [5,19]

(7) $$L={\left(\frac{k_{B}}{e}\right)}^{2}\left(\frac{\left(\lambda +\frac{7}{2}\right)F_{\lambda +\frac{5}{2}}\left(\eta \right)}{\left(\lambda +\frac{3}{2}\right)F_{\lambda +\frac{1}{2}}\left(\eta \right)}-{\left[\frac{\left(\lambda +\frac{5}{2}\right)F_{\lambda +\frac{3}{2}}\left(\eta \right)}{\left(\lambda +\frac{3}{2}\right)F_{\lambda +\frac{1}{2}}\left(\eta \right)}\right]}^{2}\right)\;\;\;\;\;\;\;\;$$

where *η* is (reduced Fermi level) calculated by utilizing the experimental Seebeck coefficient according to Equations (3) and (5) to find an accurate *L*. The calculated *L* values as a function temperature are shown in Figure 5b. It is observed that the *L* values for the 673 K, 723 K, 743 K, 773 K samples are similar, whereas the 803 K and 823 K samples show increased *L*. Then, the lattice thermal conductivity (*κ_L_*) was calculated by subtracting *κ_el_* from *κ*. The electronic thermal conductivity for the 803 K and 823 K samples are larger compared to other samples as shown in Figure 5c, which is consistent with the *σ* and *L*. The lattice thermal conductivity increased along with increase of heat treatment except for 823 K sample (Figure 5d). However, the lattice thermal conductivity decreased at a higher temperature regime for the 823 K compared to other samples, which is a result of the scattering effect, induced by carriers to phonons through the obvious increase of *n* [17]. Based on the results, the increased total thermal conductivity may be attributed to increase of grain size in the heat treatment samples.

**Figure 6:**
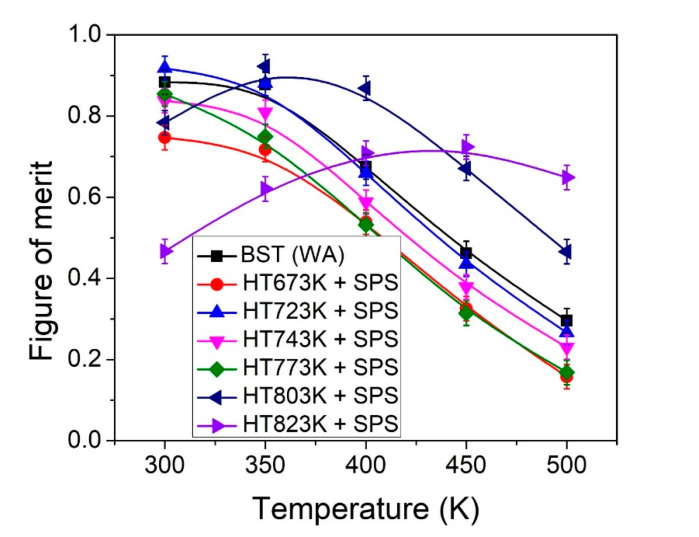
Temperature dependence of dimensionless figure of merit, ZT, for the bulk samples obtained by powder heat treatment at different temperatures and consolidation.

Figure 6 shows the temperature dependent dimensionless figure of merit (ZT) for all the bulk samples. The ZT values were decreased until 773 K of heat treatment and then increased at higher temperature (>773 K) compared to the BST sample. The peak ZT of 0.92 at 350 K was obtained for the 803 K sample. Specifically, the ZT for the 823 K sample was significantly larger at higher measurement temperature (T > 400 K), which could be useful for stable thermoelectric module performance to generate electrical power. The results suggest that the TE properties and thermal stability were degraded for the samples obtained at below 773 K of heat treatment.

## 4. Conclusions

In summary, the effect of powder heat treatment on chemical composition and thermoelectric properties of Bi_0_._5_Sb_1_._5_Te_3_ materials was systematically investigated. The results show that the Te evaporated slightly at 723 K and 743 K samples and became dominated at 803 K and 823 K, which plays a crucial role in understanding the transport properties. SEM cross-sectional microstructure of bulk samples reveals that the powder was fully dense with more random oriented grains and its size increased with increasing heat treatment temperature. The electrical conductivity was significantly improved at over 803 K heat treatment due to the remarkable improvement in hole concentration. The power factor values for 803 K and 823 K were significantly larger at T > 350 K compared to other samples. Consequently, the peak ZT of 0.92 at 350 K was obtained for the 803 K sample, which could be useful for commercial thermoelectric power generation.

## Figures and Tables

**Table 1:** X-ray fluorescence (XRF) results of trace elements in Bi_0_._5_Sb_1_._5_Te_3_ powder and bulk samples.

Sample	WA Powder	673 K	723 K	743 K	773 K	803 K	823 K
Bi	14.864 ± 0.2	15.349 ± 0.1	15.46 ± 0.1	15.6 ± 0.09	15.68 ± 0.1	15.703 ± 0.1	15.735 ± 0.1
Sb	24.374 ± 0.5	24.566 ± 0.3	24.523 ± 0.3	24.649 ± 0.4	24.849 ± 0.3	24.952 ± 0.2	25.172 ± 0.4
Te	60.557 ± 0.7	60.085 ± 0.7	60.017 ± 0.9	59.752 ± 0.8	59.471 ± 0.9	59.344 ± 1.1	59.092 ± 1.1

**Table 2:** The relative density, Seebeck coefficient (*S*), carrier concentration (*n*), mobility (*μ*), and carrier effective mass (m*) for the bulk samples.

Sample	Relative Density (%)	*S* (µV/K)	*n* (10^19^/cm^3^)	*µ* (cm^2^/Vs)	m*/m_0_
673 K	99.0 ± 0.3	237.24 ± 2.5	1.02 ± 0.05	284.78 ± 7	0.86
723 K	99.3 ± 0.17	245.01 ± 1.7	1.20 ± 0.03	293.82 ± 5	1.03
743 K	99.7 ± 0.09	245.26 ± 1.9	1.06 ± 0.07	310.9 ± 7	0.95
773 K	99.5 ± 0.19	244.40 ± 2.3	1.1 ± 0.04	307.24 ± 4	0.97
803 K	99.2 ± 0.2	178.20 ± 3.4	2.90 ± 0.07	258.54 ± 11	1.04
823 K	99.0 ± 0.5	129.60 ± 2.9	5.72 ± 0.09	171.62 ± 10	1.04

## Data Availability

The data presented in this study are available on request from the corresponding author.
